# Application of the Resazurin Cell Viability Assay to Monitor *Escherichia coli* and *Salmonella* Typhimurium Inactivation Mediated by Phages

**DOI:** 10.3390/antibiotics10080974

**Published:** 2021-08-12

**Authors:** Pedro Costa, Ana T. P. C. Gomes, Márcia Braz, Carla Pereira, Adelaide Almeida

**Affiliations:** Department of Biology and CESAM, Campus Universitário de Santiago, University of Aveiro, 3810-193 Aveiro, Portugal; pedrommrscosta@ua.pt (P.C.); ana.peixoto@ua.pt (A.T.P.C.G.); marciabraz96@ua.pt (M.B.); csgp@ua.pt (C.P.)

**Keywords:** bacteriophages, antimicrobial therapy, pathogenic bacteria, cell viability, resazurin

## Abstract

Bacterial inactivation using bacteriophages (or phages) has emerged as an effective solution for bacterial infections, but the screening methods used to evaluate the effectiveness of the phages to inactivate bacteria are not fast, reliable or precise enough. The efficiency of bacterial inactivation by phages has been evaluated by monitoring bacterial concentration either by counting colony-forming units (CFU), a laborious and time-consuming method, or by monitoring the optical density (OD), a less sensitive method. In this study, the resazurin cell viability assay was used to monitor the viability of bacteria from different genera during the inactivation by different phages, and the results were compared with the standard methods used to assess bacterial inactivation. The results showed that the resazurin colorimetric cell viability assay produces similar results to the standard method of colony-counting and giving, and also more sensitive results than the OD method. The resazurin assay can be used to quickly obtain the results of the cell viability effect profile using two different bacterial strains and several different phages at the same time, which is extremely valuable in screening studies. Moreover, this methodology is established as an effective, accurate and rapid method when compared to the ones widely used to monitor bacterial inactivation mediated by phages.

## 1. Introduction

The increased emergence of antibiotic-resistant bacteria over the last years is a major public health problem. Alternative strategies must be developed to reduce the risk of development and dissemination of microbial resistance [1,2,3]. Phage therapy is currently resurging as a potential complement/alternative to antibiotic treatment [1,2,4,5,6,7], effectively killing even multidrug-resistant bacteria [8,9,10,11,12,13,14]. With this in mind, it is essential to obtain a method that allows us to rapidly evaluate the efficiency of bacterial inactivation by phages and to test a great number of different phages at the same time to easily and rapidly preselect phages for further studies.

A great variety of methods to evaluate the efficiency of bacterial inactivation are already available and include viable plate count methods, turbidity measurements, bioluminescence assays and colorimetric test systems [15,16,17,18,19,20,21,22,23]. However, the evaluation of the efficiency of bacterial inactivation by phages is currently, and has been for decades, mainly performed by monitoring bacterial concentration during treatment time using the standard colony-forming units method [15]. Nevertheless, this method requires a relatively large number of test products [24] and is laborious and time-consuming [25,26,27], hampering its application to rapidly screen new phages and test old ones in different bacteria at the same time.

Optical density (OD) measurement has also been used in several studies as a tool to evaluate in real-time the process of bacterial inactivation by phages [28,29,30,31,32]. However, despite this method being low cost, fast and non-destructive [33], it does not allow for the reliable evaluation of bacterial viability since it may be influenced by the aggregation of the microorganisms [34], light scattering caused by anti-foam agents, dispersed gases [27], dead cells and cell debris [35] and unaccounted light scattering from dispersed inorganic salts and protein aggregates [27]. Furthermore, the turbidimetric method is only applicable in specific concentration ranges [27], and if the cell size is expected to change substantially during microbial growth (e.g., growth under antibiotics or various stresses), OD measurements are no longer suitable [36]. This makes the detection of low bacterial concentrations by OD difficult or unreliable, frequently avoiding detecting bacterial decreases and underestimating the effectiveness of phage efficiency.

The use of genetically modified bacteria with bioluminescence as a time-saving, real-time, cost-effective and simple operation for bacterial killing monitoring has been used, avoiding the laborious and time-consuming method of colony counts [14,37,38]. However, the optimum bacterial light emission conditions [39] limits the application of this method [38]. The bioluminescence signal of bacteria depends on temperature, oxygen concentration [38,39] and the detection limit of the luminometer recorder [40]. These factors make this methodology limited to bacterial inactivation by phages, which is, in general, long. Moreover, this method can only be used for genetically modified or naturally bioluminescent bacteria. Consequently, this approach is more adequate to study, for instance, the phage-host interaction or the impact of physical and chemical conditions on the efficiency of phages to inactivate bacteria [38,39].

The MTT (3-(4,5-dimethylthiazol-2-yl)-2,5-diphenyltetrazolium bromide) tetrazolium assay is a popular colorimetric assay used to estimate the metabolic activity of living cells [41]. Originally devised to be used in eukaryotic cells lines [42,43], it was later applied in bacteria and fungi [41]. It consists of the spectrophotometric quantification of the intense purple-blue color of formazan, an enzymatic reduction of the lightly colored tetrazolium salt. This colorimetric assay has already been used to determine phage screening [22], assess bacterial viability after phage treatment [19,20,21] and monitor biofilm inactivation by phages [23]. However, the absorption wavelength of tetrazolium salt reduction products is between 500 and 600 nm [41], which coincides with the wavelength at which the bacterial optical density is read. This factor can lead to the misinterpretation of the results since both absorbances of bacterial turbidity and the purple-blue color of formazan will be read in the spectrophotometer. Other variants, like the XTT (2,3-bis(2-methoxy-4-nitro-5-sulfophenyl)-5-carboxanilide-2*H*-tetrazolium), can be used to overcome some of the MTT drawbacks by being more soluble and having a different absorption wavelength (450–500 nm) [41].

Resazurin cell viability assays or Alamar Blue^TM^ assays have been widely used over the past 50 years in studies on cell viability and cytotoxicity in a range of biological and environmental systems and have been applied to monitor antimicrobial susceptibility and microbial biofilms viability [24,26,44,45]. Resazurin is a cell-permeable redox indicator that can be used to monitor viable cell numbers with protocols similar to those of the tetrazolium compounds. This non-fluorescent blue dye is converted into pink-fluorescent resorufin in the presence of metabolically active cells [24,46]. NADPH dehydrogenase is probably responsible for the transference of electrons from NADPH + H+ to resazurin, which is reduced to resorufin. This conversion can be detected through the visual observation of its pink color or absorbance readings of the ratio of resorufin/resazurin at 570/600. Additionally, unlike other colorimetric methods, it can be detected by fluorimetry or by measuring the fluorescence of the resorufin at an excitation maximum of 530–570 nm and an emission maximum of 580–590 nm [47]. The access of cell viability through fluorescence offers a huge advantage when compared to the other cell viability assays and the monitoring of OD since it overcomes overlapped results achieved by bacterial turbidity. This method has also some more advantages, including speed, reliability, sensitivity, safety and cost [24,26,46,48] (approximately 216 to 230 € or 230 to 270 USD per 10 g). In addition, it keeps cells intact, which allows for other parallel analyses [24]. This colorimetric assay has already been used to determine phage host range [49] and assess bacterial viability after phage treatment [22,45,49,50,51,52,53]. However, no comparison of the method’s reliability with the most frequently used methods, viable plate count method and optical density measurement, was done to evaluate the effectiveness of bacterial inactivation by phages.

The main goal of this work was to evaluate the reliability of the resazurin cell viability assays to be used as a fast and reliable screening technique to preselect already isolated phages based on the bacterial killing curves resulting from bacterial inactivation by phages and to compare those results with the standard methods used to access bacterial inactivation. For this, we monitored the viability of two different bacterial cultures, *Escherichia coli* and *Salmonella enterica* serovar Typhimurium (ATCC 13311), in the presence of two previously isolated and characterized phages, ELY-1 (T4-like phage with double-stranded DNA of the family *Myoviridae*) and phSE-5 (double-stranded DNA phage of the family *Siphoviridae*) [1,38], along with six other phages (phEc1, phEc2, phEc3, phST1, phST2 and phST3) recently isolated from sewage water samples. The results obtained were compared with the ones achieved with the standard method of counting colony-forming units and the OD measurement.

## 2. Results

### 2.1. Relation between CFU, OD 600 nm and RFU

**Figure 1:**
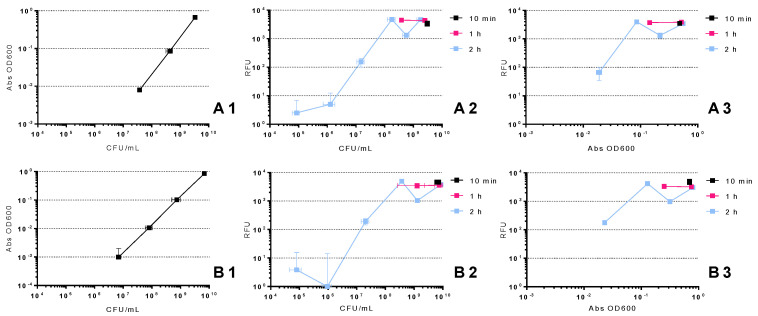
Relationship between optical density and viable counts (**1**), fluorescence after 10 min, 1 h and 2 h of incubation and viable counts (**2**) and fluorescence after 10 min, 1 h and 2 h of incubation and optical density (**3**) of overnight cultures of *E. coli* (**A**) and *S.* Typhimurium (**B**). Optical density is expressed in absorbance (Abs OD 600 nm), fluorescence in relative fluorescence units (RFU) and viable counts in colony-forming units per milliliter (CFU/mL). The values are expressed as the means of three independent experiments; *error bars* indicate the standard deviation. Some values are missing because they go below the method’s lower detection limit and would go to a negative Y value, and negative numbers cannot be shown on a logarithmic axis.

In order to compare the relation, mainly the minimum and maximum limits, between the standard test of colony-forming unit counts, optical density and resazurin methods, relationship curves for both bacteria were constructed. Those were established between absorbance at 600 nm, relative fluorescent units (RFU) and viable counts of a growing culture of *E. coli* and *S.* Typhimurium and are presented in Figure 1. The colony-forming units (CFU) represent the bacterial number per milliliter obtained with the standard culture method, the Abs at 600 nm represent the turbidity measured in the optical density method and RFU results reflect cell viability obtained in the resazurin method.

When assessing the relation between the absorbance and viable counts of both *E. coli* and *S.* Typhimurium (Figure 1(A1,B1)), no absorbance values at 600 nm were detected below 7 log CFU/mL. The maximum absorbance values at 600 nm were detected at 10 log CFU/mL.

When assessing this relationship for both *E. coli* and *S.* Typhimurium (Figure 1(A2,B2)), no RFU values were detected below 5 log CFU/mL, and the maximum RFU values detected occurred at approximately 8 and 10 log CFU/mL. Since the resazurin method employed a maximum detection limit of 4 log RFU, during the assays, 10 min and 1 h incubation periods were used to obtain the maximum relative fluorescence for 9 and 10 log CFU/mL.

When assessing the relationship between fluorescence and absorbance in both *E. coli* and *S.* Typhimurium (Figure 1(A3,B3)), no values were detected below 2 log RFU and −2 log Abs OD 600 nm. The maximum values observed were detected at 4 log RFU.

### 2.2. Bacterial Killing Curves

#### 2.2.1. Effect of *E. coli* Phages in the Inactivation of *E. coli*

Since phages can infect and inactivate bacteria different from their hosts [54], and in order to further evaluate the effectiveness of the different methods to assess the bacterial inactivation resulting from different phages, besides the previously isolated phage ELY-1, three other *E. coli* phages isolated using a different strain of *E. coli* as host were used.

**Figure 2:**
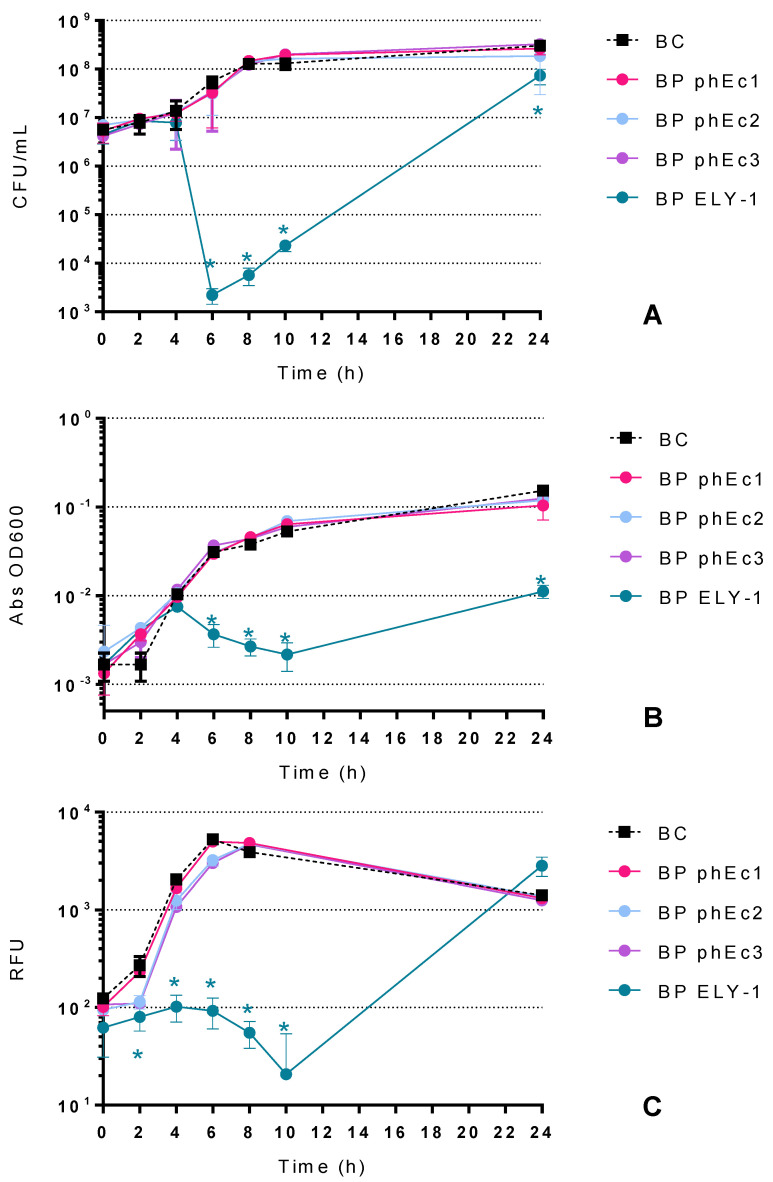
Inactivation of *E. coli* by four *E. coli* phages (phEc1, phEc2, phEc3 and ELY-1) at a multiplicity of infection (MOI) of 10 for 24 h. Bacterial concentration of *E. coli* through colony-counting (**A**), optical density (**B**) and cell viability (**C**) methods: BC-Bacteria Control; BP-Bacteria plus Phage. Values represent the mean of three independent assays; error bars represent the standard deviation. * *p* < 0.05 (relative to the respective control).

From the four *E. coli* phages, only phage ELY-1 inactivated the bacterium, achieving a maximum inactivation of 4.4 log CFU/mL after 6 h of incubation (ANOVA, *p* < 0.05, Figure 2A) when compared with the bacterial control (BC). Bacterial density in the BC increased by 1.7 log CFU/mL (ANOVA, *p* < 0.05, Figure 2A) after 24 h of incubation.

When the growth of *E. coli* was monitored by OD 600 nm (Figure 2B), no significant differences were observed in the *E. coli* growth profile in the first 4 h of incubation with phage ELY-1 compared to the BC. Only after 6 h of incubation was it possible to observe a decrease in OD 600 nm in the sample with ELY-1 (ANOVA, *p* < 0.05, Figure 2B). For phages phEc1, phEc2 and phEc3, no significant differences were observed during the experiments compared to the BC.

When the inactivation profile of *E. coli* was monitored by the resazurin viability assay (Figure 2C), it was also observed that only phage ELY-1 significantly affected the viability of *E. coli* after 2 h of incubation (ANOVA, *p* < 0.05), with a maximum decrease of cell viability after 10 h of incubation (ANOVA, *p* < 0.05).

#### 2.2.2. Effect of *S.* Typhimurium Phages in the Inactivation of *S.* Typhimurium 

**Figure 3:**
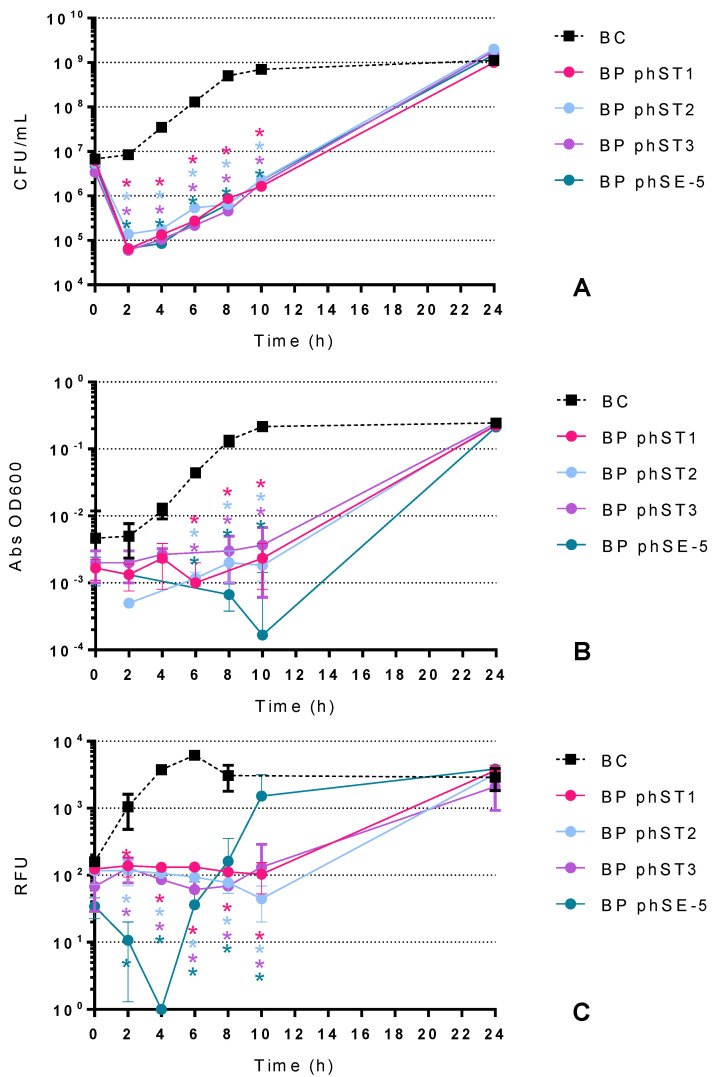
Inactivation of *S.* Typhimurium by four *S.* Typhimurium phages (phST1, phST2, phST3 and phSE-5) at an MOI of 10 for 24 h. Bacterial concentration of *S.* Typhimurium through colony-counting (**A**), optical density (**B**) and cell viability (**C**) methods: BC-Bacteria Control; BP-Bacteria plus Phage. Values represent the mean of three independent assays; error bars represent the standard deviation. * *p* < 0.05 (relative to the respective control). Some values are missing because they go below the method’s lower detection limit and would go to a negative Y value, and negative numbers cannot be shown on a logarithmic axis.

The maximum inactivation with phages phST1, phST2, phST3 and phSE-5 was 2.8, 2.9, 3.0 and 2.9 log CFU/mL achieved after 8 h of incubation (ANOVA, *p* < 0.05, Figure 3A), respectively, when compared to the BC. Bacterial density in the BC increased by 2.2 log CFU/mL (ANOVA, *p* < 0.05, Figure 3A) during the 24 h of incubation.

When the growth of *S.* Typhimurium was monitored by OD 600 nm (Figure 3B), no significant differences were observed in the *S.* Typhimurium growth profile in the first 4 h of incubation for any of the four phages tested compared to the BC. Only after 6 h of incubation was it possible to observe a decrease in OD 600 nm in all samples and BC (ANOVA, *p* < 0.05, Figure 3B). After 10 h of incubation, the rate of bacterial inactivation with phage phSE-5 was significantly higher (ANOVA, *p* < 0.05) than the ones obtained with phages phST1, phST2 or phST3.

When the inactivation profile of *S.* Typhimurium was monitored by the resazurin viability assay (Figure 3C), it was observed that all four phages significantly affected the viability of *S.* Typhimurium after 2 h of incubation (ANOVA, *p* < 0.05). However, the maximum decrease of cell viability occurred after 4 h of incubation for phage phSE-5 and after 6 h of incubation (ANOVA, *p* < 0.05) for phages phST1, phST2 and phST3.

### 2.3. Phage Concentration

**Figure 4:**
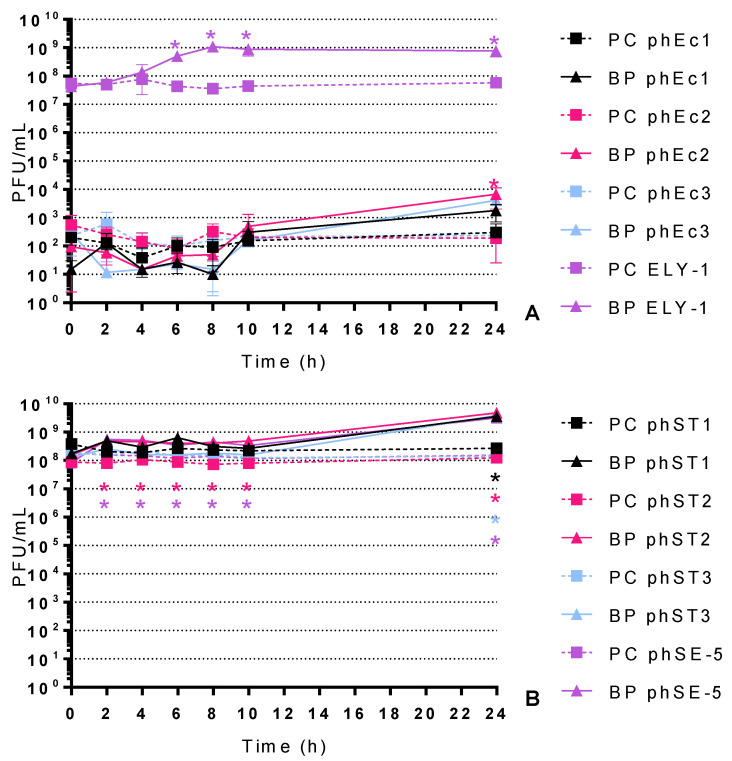
Phage concentration of *E. coli* phages (phEc1, phEc2, phEc3 and ELY-1) in the presence of *E. coli* (**A**) and *S.* Typhimurium phages (phST1, phST2, phST3 and phSE-5) in the presence of *S.* Typhimurium (**B**) at an MOI of 10 for 24 h: PC-Phage Control; BP-Bacteria plus Phage. Values represent the mean of three independent assays; error bars represent the standard deviation. * *p* < 0.05 (relative to the respective control).

The phage concentration was also monitored during the assays (Figure 4). Since the resazurin method only evaluates bacterial activity, phage concentration was solely monitored through the direct visual detection of plaque-forming units (PFU).

The phage controls (PC) remained constant during the 24 h of the experiments (ANOVA, *p* > 0.05, Figure 2).

In the presence of *E. coli*, the survival factor of phages phEc1 and ELY-1 concentration increased up to 2.1 and 1.1 log PFU/mL (ANOVA, *p* < 0.05, Figure 4A) after 24 and 6 h of incubation, respectively. Phages phEc1, phEc2 and phEc3 started with a lower phage concentration than phage ELY-1.

In the presence of *S.* Typhimurium, phages phST1, phST2, phST3 and phSE-5’s concentration factors increased up to 1.1, 1.6, 1.4 and 1.3 log PFU/mL (ANOVA, *p* < 0.05, Figure 4B) after 24 h of incubation, respectively.

## 3. Discussion

Bacterial inactivation using phages has emerged as an effective solution for bacterial infections. Although the potential of this therapy is enormous, the most common methods to evaluate and assess the effectiveness of phage efficiency, namely, colony-counting [15,16,18] and OD [28,29,30,31,32] methods, are either too time-consuming [25,26,27] or don’t have enough precision [27,36] for screening purposes. As such, this study aimed to determine if the resazurin assay in microplates could be used to screen and preselect several already isolated *E. coli* (ATCC 25922) and *S.* Typhimurium (ATCC 13311) phages at the same time based on their bacterial inactivation profile and to compare those results with the standard methods used to access bacterial inactivation. Moreover, it is intended to use this methodology to screen the bacterial inactivation in the presence of such phages before pursuing more deep characterization studies. For this, we monitored the inactivation of two bacteria of different genera (*E. coli* and *S.* Typhimurium) by two previously isolated and characterized phages, ELY-1 and phSE-5 [1,38], and six recently isolated phages (phEc1, phEc2, phEc3, phST1, phST2 and phST3) and compared the results obtained with those achieved with the standard colony-counting and OD methods. The results showed that the resazurin method can effectively provide clear inactivation profiles for the different phages for both *E. coli* and *S.* Typhimurium quicker than the standard colony-counting method and with more accuracy than the OD method. This data is very important for screening phages based on their bacterial inactivation profile.

### 3.1. Colony-Counting Method

Since eight different phages and two bacteria were used, it was possible to observe different growth rates and killing curves through the use of the colony-counting method. This method has the main advantages of well-standardized protocols and usually higher sensitivity compared to other methods [55]. Although very precise, like other authors suggested [24,25,26,27], colony-counting in solid medium is too complex, laborious and time-consuming to test more than two samples with their correspondent control at the same time. This is more evident when considering the time expended with serial dilutions and plating when multiple samples are being studied at once. Moreover, the time needed for the results to be known depends on the bacterial growth rate, which can take between 12 and 24 h. As such, it is not ideal as a fast-screening method to evaluate the inactivation profile of newly isolated phages or to test a specific phage on several other bacterial strains at once.

### 3.2. Optical-Density Method

OD measurement is a very fast method to monitor bacterial growth/inactivation in real-time, but it lacks accuracy. As seen in the results, when bacterial inactivation occurs in the first 4 h of the assays, even with high concentrations of bacteria (7 log CFU/mL), the absorbance is too close to the method’s lower detection limit (Figure 1) to observe any early inactivation and to efficiently discriminate the effectiveness of the different phages (Figure 3B), mainly when the bacterial inactivation starts and when it reaches its real maximum. Some authors suggested that OD measurements are not suitable when substantial changes in cell size are expected to occur during microbial growth [36] and that it is only applicable in specific concentration ranges [27]. As such, the OD method is only suitable in cases where the bacterial inactivation occurs at a later stage (Figure 2) or when an even greater bacterial concentration is used. Nevertheless, that will require a higher concentration of phage suspension, which is sometimes hard to produce.

### 3.3. Resazurin Method

The results with the resazurin cell viability method showed similar bacterial inactivation profiles to the ones observed in the colony-counting methodology for both *E. coli* and *S.* Typhimurium (Figure 2 and Figure 3, respectively), demonstrating that the resazurin microplate method is as accurate as the plate colony-count technique. Despite the inactivation of *E. coli* by phage ELY-1 monitored by colony-forming units not showing a perfect match with the results achieved with the resazurin cell viability assay in the first 4 h of incubation, in general, all profiles are similar. It cannot be neglected that resazurin assays measure the cell viability of bacteria, which does not mean that when the fluorescence decreased the bacteria were inactivated, but only affected, showing a decrease in their viability. These small variations on the bacterial viability cannot be detected by colony-counting methodology. Similar findings were observed by Toté et al. [24], where the authors developed a resazurin microplate method for the evaluation of the antimicrobial activity of antiseptics and disinfectants on several different bacteria and reported that this method was as precise as the plate count method, showing similar detection limits.

#### 3.3.1. Resazurin Method—Advantages

Since the resazurin method is fast, not laborious and uses the modified cell viability method [56,57] to monitor bacteria, it was possible to study several different conjugations of bacteria and phages in duplicate in the same experiment and for the results to be known only 2 h after the sample was taken. This high-throughput setup enabled the measurement of a whole range of different bacteria-phage combinations at once, with each sample in duplicate for better accuracy. In the absence of phages, the bacterial growth was highly consistent for all samples during the assay. Moreover, the resazurin method has a lower detection limit than the OD method (5 and 7 log CFU/mL, respectively, as shown in Figure 1), which makes it ideal to observe any early inactivation and to efficiently discriminate the effectiveness of the different phages. Furthermore, the nontoxicity of resazurin is advantageous since allows for dynamic measurements [16,24,56,58,59].

#### 3.3.2. Resazurin Method—Disadvantages

Nevertheless, some points needed to be addressed. The resazurin reduction reaction is rapid in high bacterial concentrations (in some cases occurring immediately after the addition of the compound), corresponding to cell viability values (fluorescence) above the upper detection limit of the method (approximately 10 log CFU/mL) (Figure 1). That is, if the cell count is too high or incubated for too long, an extensive reduction of resazurin leading to the final nonfluorescent product (hydroresorufin) may occur (bleaching effect), and an underestimation of cellular activity may be obtained [60]. However, this can be surpassed by decreasing the incubation period (Figure 1). Another point is in regard to the initial bacterial concentration, where the assays must start with an estimated bacterial concentration of at least 6 to 7 log CFU/mL (Figure 1) since the lytic effects of some phages can result in bacterial counts below the lower detection limit (approximately 5 log CFU/mL) at initial times. Third, the resazurin incubation time was limited to 2 h to ensure synchronized growth for all samples; hence, potential fluorescence at later time points and post lytic effects could not be studied. Finally, since the resazurin assay only measures the metabolic activity of bacteria through aerobic respiration, it is not ideal to evaluate the phage killing curves of anaerobic bacteria. Moreover, since there are phages that infect and kill bacteria in both aerobic and anaerobic conditions, further studies are needed to understand if this method can be affected by interactions between bacteria and phages, such as adsorption or infection without lysis. Furthermore, this trait makes it unsuitable to discriminate between phage particles or any other bacterial inactivation agent, as it happens with the colony-counting methodology.

### 3.4. Phage Concentration

To better understand the bacterial inactivation, phage concentration was also monitored during the assays (Figure 4). As seen in the results, the newly isolated *E. coli* phages started with a lower concentration than the previously isolated phage ELY-1 (Figure 4A) because they have a lower efficiency of plating, which is expected from phages that have different hosts. However, unlike in a previous study [55], no bacterial inactivation was detected (Figure 2A), despite infecting the bacterium. As such, further studies are needed to understand the dynamic between these phages and *E. coli*. Relative to *S.* Typhimurium, all tested phages multiplied in the presence of the host during the experiment (Figure 4B) and efficiently inactivated the bacterium (Figure 3A).

### 3.5. Other Applications of the Resazurin Method with Phages

Like other studies [24,49], this study demonstrated that the resazurin microplate method is faster and as accurate as the colony-count technique and is more precise and has a lower minimum detection limit than the OD method. As such, this fluorometric method is a good alternative to monitor the concentration of different bacteria during their inactivation by several phages at the same time and, consequently, is a promising technique to screen and preselect new or known phages by their inactivation profile.

Other colorimetric methods were successfully used in phage screening [22,49,50,51,52,53]. Some authors used the tetrazolium-based assay to screen 98 single *Acinetobacter baumannii*-specific lytic phages to inactivate *A. baumannii* [22], others used the resazurin assay to assess the cell activity of *Pectobacterium atrosepticum* with CRISPR-Cas immunity upon infection with the virulent phages [52], others used the resazurin assay as a fast screening assay to preselect *Dickeya solani* and *Pectobacterium parmentieri* Tn5 mutants in genes coding for proteins used by lytic phages φD5 and φA38 as receptors [49] and others used the resazurin assay to detect for the presence of phages specific to *Streptococcus diacetilactis* in cottage cheese samples [61]. However, these studies only tested the phages’ ability to inactivate or not inactivate the targeted bacterium. One study used the resazurin method to assess *Salmonella* inactivation 1 h after incubation [62]. Some studies used the resazurin assay to assess if the biofilm cell metabolic activity was affected or not by the phage addition after a specific amount of time [50,51,53,63]. One study assessed *Klebsiella* biofilm viability 2 h after different phages were added [63], M. Al-Zubidi and colleagues [51] assessed the phage SHEF2 ability to clear *Enterococcus faecalis* biofilms 3 h after incubation, Mendes et al. [53] and Haines et al. [64] assessed the susceptibility of different bacterial biofilms to phages 4 and 24 h after incubation and Topka and colleagues [50] assessed the metabolic activity/viability of *E. coli* biofilm cells with different MOI of phage vB_EcoS-95 for 150 min, 4 h after phage incubation.

However, to our knowledge, this is the first study that uses the resazurin method to obtain *E. coli and S.* Typhimurium inactivation curves by several different phages at once over a longer period and is the first to compare those results with the standard methods used to access bacterial inactivation.

## 4. Materials and Methods

### 4.1. Bacterial Strains and Growth Conditions

The bacterial strains recombinant bioluminescent *E. coli* [39], *E. coli* FDA strain Seattle 1946 [DSM 1103, NCIB 12210] (ATCC 25922) and *S.* Typhimurium NCTC 74 (ATCC 13311) were used in this study as phage hosts. All bacteria were grown in Tryptic Soy Broth (TSB; Liofilchem, Roseto degli Abruzzi, Italy). The fresh plate bacterial cultures were maintained in Tryptic Soy Agar medium (TSA; Liofilchem, Roseto degli Abruzzi, Italy) at 4 °C. Before each assay, one isolated colony was aseptically transferred to 30 mL of TSB and grown overnight at 25 °C at 120 rpm stirring. An aliquot of this culture (100 µL) was transferred to 10 mL of fresh TSB medium and grown overnight at 25 °C to reach an optical density (O.D. 600 nm) of 0.8 (HaloDB-20; DynamicaScientific, Livingston, UK), corresponding to about 10^9^ cells/mL.

### 4.2. Relation between CFU, OD 600 nm and RFU

To assess the relation between CFU, RFU and absorbance, overnight cultures of *E. coli* and *S.* Typhimurium were serially diluted (10^−1^–10^−5^) in TSB medium.

In order to determine the relationship between fluorescence and the other two methods, three incubation periods at 37 °C were considered (10 min, 1 h and 2 h) after the sample was taken. To ensure a synchronized growth for all samples, a 2 h incubation period was established, since it was the period that had the best relation between fluorescence, viable counts and absorbance.

For the resazurin cell viability method, 100 μL of the non-diluted and diluted aliquots were added to black-opaque 96 well plates with 20 μL of resazurin (0.15 mg/mL). The samples were then incubated at 37 °C in the dark for 10 min, 1 h and 2 h to find the best relation between fluorescence and viable counts. A 2 h incubation period was established to ensure a synchronized growth during the assays. The ability of bacteria to reduce resazurin into fluorescent resorufin was measured by spectrofluorimetry using a Synergy HT Pro microplate reader (BioTek Instruments, Winooski, VT, USA) with GEN5 software, with excitation set at 550 nm and emission set at 590 nm. The values obtained from the readings were expressed as RFU. After each measurement, 100 μL of each dilution were serial diluted and drop-plated in TSA medium. The plates were incubated at 25 °C, and the bacterial concentration (CFU/mL) was calculated after 24 h of incubation.

For the OD method, 100 μL of the non-diluted and diluted aliquots were added to clear 96 well sterile polystyrene plates. The culture turbidity was measured as a proxy for bacterial density by spectrophotometry using a Multiskan™ FC Microplate Photometer (Thermo Fisher Scientific Inc., Waltham, MA, USA) set at 600 nm and, simultaneously, 100 μL of each dilution were serial diluted and drop-plated in TSA medium. The plates were incubated at 25 °C and the bacterial concentration (CFU/mL) was calculated after 24 h of incubation.

Both experiments were done in triplicate and the results were averaged.

### 4.3. Phage Isolation, Purification and Preparation

The *E. coli* phages phEc1, phEc2 and phEc3 and the *S.* Typhimurium phages phST1, phST2 and phST3 were isolated from sewage water samples collected in the sewage network of Aveiro (SIMRIA Multi Sanitation System of Ria de Aveiro—station EEIS9), all gathered at different times. One hundred milliliters of water were filtered through 0.45 µm pore size polycarbonate membranes (Millipore; Bedford, MA, USA). The filtered water was added to 100 mL of a twice concentrated TSB medium with 1 mL of a fresh culture of the hosts, *E. coli* (ATCC 25922) and *S*. Typhimurium (ATCC 13311). The mixtures were incubated at 25 °C for 18 h at 80 rpm and afterwards centrifuged at 10.000× *g* for 10 min at 4 °C and filtered through a polyethersulphate layer with a 0.22 µm pore size (Merck-Millipore; Darmstadt, Germany). The suspensions were stored at 4 °C and the titer was determined by the double-layer agar method [15]. Successive dilutions of the suspensions were done in phosphatecradled saline (PBS) [137 mmol^− 1^ NaCl (Sigma; St. Louis, MO, USA), 8.1 mmol^− 1^ Na_2_HPO_4_·2H_2_O (Sigma; St. Louis, MO, USA), 2.7 mmol^− 1^ KCl (Sigma; St. Louis, MO, USA) and 1.76 mmol^− 1^ KH_2_PO_4_ (Sigma; St. Louis, MO, USA), pH 7.4). 500 µL of each dilution, along with 200 µL of fresh bacterial culture (*E. coli* (ATCC 25922) or *S.* Typhimurium (ATCC 13311)), were mixed in 5 mL of TSB 0.6% top agar layer [30 g/L TSB (Liofilchem; Roseto degli Abruzzi, Italy), 6 g/L agar (Liofilchem; Roseto degli Abruzzi, Italy), 0.12 g/L MgSO_4_ (Sigma; St. Louis, MO, USA) and 0.05 g/L CaCl_2_ (Sigma; St. Louis, MO, USA), pH 7.4] and poured over a TSA plate. Plates were incubated at 25 °C and observed for the presence of lytic plaques after 12 h. One single plaque was selected from the agar and added to TSB medium with a fresh culture of the host. The sample was centrifuged, being the supernatant used as a phage source for a second isolation procedure. Three successive single-plaque isolation cycles were performed to acquire pure phage stocks. All lysates were centrifuged at 10,000× *g* for 10 min at 4 °C to remove bacteria or bacterial debris. The phage suspensions were kept at 4 °C. Phage stocks were prepared from the phage suspensions purified in SM buffer (0.1 M NaCl (Sigma-Aldrich; St. Louis, MO, USA), 20 mM Tris-HCl (Sigma; St. Louis, MO, USA) and 8 mM MgSO_4_ (Sigma; St. Louis, MO, USA), pH 7.5), using *E. coli* (ATCC 25922) and *S.* Typhimurium (ATCC 13311) as hosts. After incubation, the stock culture of *E. coli* and *S.* Typhimurium in the exponential growth phase was centrifuged at 10,000× *g* for 10 min, and the pellet was resuspended in 30 mL of SM buffer. Then, three hundred microliters of the phage stock were added to 30 mL of SM buffer with bacteria. The phage stocks were incubated at 25 °C under an orbital shaking set at 50 rpm. The lysate was centrifuged at 10.000× *g* for 10 min at 4 °C and the supernatant was filtered through a polyethersulphate membrane with a 0.22 µm pore size (Merck-Millipore; Darmstadt, Germany). The phage suspension was stored at 4 °C and the titer was determined via the double-layer agar method [15], as described above. The plates were incubated at 25 °C for 12 h and the number of lysis plaques was counted. The results were expressed as plaque-forming units per milliliter (PFU/mL).

Phages ELY-1 and phSE-5 were isolated in previous works from water samples collected from the Corte das Freiras aquaculture [38] and sewage network of Aveiro (station EEIS9 of SIMRIA Multi Sanitation System of Ria de Aveiro) [1], respectively. Phage ELY-1 (accession number KC755108) was identified as a T4-like phage with double-stranded DNA of the order Caudovirales, family *Myoviridae*, with 95% of homology with the Enterobacteriaceae phage vB_EcoMVR7 (accession number HM563683) [38]. Phage phSE-5 (accession number KX015771) was identified as a double-stranded DNA phage of the order Caudovirales, family *Siphoviridae*, with 94% homology with the *Siphoviridae* phages, TLS (accession number AY308796.1) and Salmonella phage FSL SP-126 (accession number KC139513.1) [1].

Phage suspensions were prepared from the phage stock prepared previously in SM buffer [0.1 M NaCl (Sigma), 8 mM MgSO_4_ (Sigma), 20 mM Tris-HCl (Sigma) and 2% (*w/v*) gelatin, pH 7.5]. Three hundred microliters of the phage stock were added to thirty millilitres of SM buffer and 1 mL of *E. coli* or *S.* Typhimurium in an exponential growth phase. The suspension was grown overnight and incubated at 25 °C at 50 rpm. The lysates were incubated with chloroform (final volume of 1%) for 1 h at 120 rpm. After incubation, the lysate was centrifuged at 13.000 rpm for 10 min at 4 °C to remove intact bacteria or bacterial debris. Phage suspension was stored at 4 °C and the titre was determined using the double-layer agar method [15].

### 4.4. Bacterial Killing Curves

The bacterial inactivation was quantified in the presence and absence of each phage using three methods: (i) determining bacterial concentration by the colony-counting method, (ii) determining population growth rate using the optical density method (O.D. 600 nm) and (iii) determining cell viability using the resazurin method.

Bacterial inactivation was determined using single phage suspensions at a multiplicity of infection (MOI) of 10, with an initial bacterial concentration of approximately 7 log CFU/mL and an initial phage concentration of approximately 8 log PFU/mL. In the assays with the phages, it was used as host the bacterium *S*. Typhimurium or *E. coli*. For each assay, two control samples were included: the bacterial control (BC) and the phage control (PC). The bacterial control was not inoculated with phages and the phage controls were only inoculated with phages. Controls and test samples were incubated exactly in the same conditions. The aliquots of test samples and controls were collected at time 0 and after 2, 4, 6, 8, 10 and 24 h of incubation. The bacterial concentration was determined by the pour and drop-plating methods in duplicate in TSA medium. The plates were incubated at 25 °C and the bacterial concentration was calculated after 24 h incubation and expressed as CFU/mL. The phage titre was determined in duplicate through the double-agar layer method [15] after an incubation period of 12 h at 25 °C. The results were expressed as plaque-forming units per millitre (PFU/mL).

For the OD method, 100 μL of the test samples and controls were collected at time 0 and after 2, 4, 6, 8, 10 and 24 h into clear 96 well sterile polystyrene plates. The culture turbidity was measured as a proxy for bacterial density by spectrophotometry using a Multiskan™ FC Microplate Photometer (Thermo Fisher Scientific Inc.; Waltham, MA, USA) set at 600 nm.

In the resazurin cell viability method, 100 μL of the test samples and controls were collected at time 0 and after 2, 4, 6, 8, 10 and 24 h into black-opaque 96 well plates and 20 μL of resazurin (0.15 mg/mL) were added to each well. The samples were incubated at 37 °C in the dark for 2 h to obtain the maximum RFU. The ability of bacteria to reduce resazurin into fluorescent resorufin was measured by spectrofluorimetry using a Synergy HT Pro microplate reader (BioTek Instruments; Winooski, VT, USA) with GEN5 software (BioTek Instruments; Winooski, VT, USA), with excitation set at 550 nm and emission set at 590 nm. At 24 h, the incubation time required for a maximum RFU was only 10 min. The results were expressed in RFU. Three independent experiments were performed for each method and condition.

### 4.5. Statistical Analysis

The statistical analysis of data was performed using GraphPad Prism 7.04 software (San Diego, CA, USA). Normal distributions were checked by the Kolmogorov-Smirnov test and the homogeneity of variances was assessed by Levene’s test. Whenever significance was accepted at *p* < 0.05, Tukey’s and Sidak’s multiple comparison tests were used for the pairwise comparison of the means. The significance of the bacterial and viral concentrations between treatments and along the experiments was tested using the two-way analysis of variance (ANOVA) and the Bonferroni post-hoc test. For different treatments, the significance of differences was evaluated by comparing the results obtained in the test samples with the results obtained for the correspondent control samples, for the different times.

## 5. Conclusions

This work has shown that resazurin cell viability assay can effectively assess bacterial inactivation by several different phages at the same time. The decrease profiles given by the resazurin method are similar to those given by the colony counting method, making this colorimetric method a promising alternative to OD measurements as a fast and precise method to assess bacterial inactivation by phages. This method can provide a lower detection limit than the OD method and be used to assess the inactivation of different strains by several different phages at the same time, which is extremely valuable in screening studies and the preselection of phages.

## Data Availability

Data is contained within the article.
